# Complete Genome Sequence of Zika Virus Isolated in Mexico, 2016

**DOI:** 10.1128/genomeA.00750-16

**Published:** 2016-08-04

**Authors:** José Alberto Díaz-Quiñonez, Rocío Peña-Alonso, Edgar Mendieta-Condado, Fabiola Garcés-Ayala, Elizabeth González-Durán, Noé Escobar-Escamilla, Mauricio Vázquez-Pichardo, María de la Luz Torres-Rodríguez, Alma Núñez-León, Belem Torres-Longoria, Irma López-Martínez, Cuitláhuac Ruíz-Matus, Pablo Kuri-Morales, José Ernesto Ramírez-González

**Affiliations:** a Instituto de Diagnóstico y Referencia Epidemiológicos (InDRE) Dr. Manuel Martínez Báez, Mexico City, Mexico; b Facultad de Medicina, UNAM, Mexico City, Mexico; c Dirección General de Epidemiología, Mexico City, Mexico; d Subsecretaría de Prevención y Promoción de la Salud, Mexico City, Mexico; e Escuela Nacional de Ciencias Biológicas, IPN, Mexico City, Mexico

## Abstract

Zika virus belongs to the genus *Flavivirus*, and its spread remains an international public health emergency. In this report, we describe the obtainment and molecular characterization of a complete viral genome through the direct metagenomic analysis from saliva from an autochthonous transmission case in Mexico.

## GENOME ANNOUNCEMENT

Zika virus (ZIKV) is an emerging arbovirus transmitted by *Aedes* mosquitoes and belongs to the genus *Flaviviridae*. Most often, signs and symptoms are maculopapular rash, fever, arthralgia, myalgia, headache, and conjunctivitis; edema, sore throat, cough, and vomiting occur less frequently (1).

Here, we report the complete genome sequence of a Zika virus strain MEX/InDRE/Sm/2016 directly from the saliva of an autochthonous case. Strain MEX/InDRE/Sm/2016 was identified in the saliva of a 22-year-old woman. Viral RNA was isolated using the QIAamp viral RNA minikit and selectively purified with the RiboMinus eukaryote kit. A single-end library was generated and sequenced using the Ion Torrent platform, and 946,499 reads for MEX/InDRE/Sm/2016 were obtained. The complete genome was assembled using Tmap version 4.2.18, using the sequence of Zika virus strain MRS_OPY_Martinique_PaRi_2015 as a mapping reference (accession no. KU647676.1).

A consensus sequence was obtained, with a total length of 10,617 bp and an average coverage of 134× The sequence was annotated and submitted using the NCBI BankIt tool. The obtained sequence contains a single open reading frame (ORF) that encodes a polyprotein that is cleaved into the following structural proteins: capsid (C), premembrane (Pr), membrane (M), and envelope (E), and 8 nonstructural proteins (NS1, NS2A, NS2B, NS3, NS4A, 2K, NS4B, and NS5).

Maximum-likelihood phylogenetic analysis on a genomic scale using the Tamura-Nei substitution model revealed that the strain belongs to the Asian lineage and is related to strains identified in America during the virus dispersion from Southeast Asia. The closest evolutionary relationship was established with the strain causing an outbreak in Martinique at the end of 2015 (2), forming a clade in which the strains identified in Colombia, Panama, and the virus sequenced from the amniotic fluid of a fetus with microcephaly in Brazil (3) in the same season are included. An *in silico* mutation analysis in the different ORFs revealed different amino acid changes: in particular, our sequence presented two mutations observed in the Asian lineage isolated only in the Americas and French Polynesia, those of S17N in the propeptide region of the capsid protein, and M114V in the NS5 protein. The molecular markers that defined relationship with the strain from Martinique were V80T in NS2A and T833A in NS5. Additionally, two amino acid changes were observed only in Zika virus genome from Mexico: A106E in NS3 and H289Q in the NS5 protein.

### Nucleotide sequence accession number.

The assembled complete genome sequence of MEX/InDRE/Sm/2016 was submitted to GenBank with accession no. KU922960.
